# The APAC Score: A Novel and Highly Performant Serological Tool for Early Diagnosis of Hepatocellular Carcinoma in Patients with Liver Cirrhosis

**DOI:** 10.3390/jcm10153392

**Published:** 2021-07-30

**Authors:** Joeri Lambrecht, Mustafa Porsch-Özçürümez, Jan Best, Fabian Jost-Brinkmann, Christoph Roderburg, Münevver Demir, Frank Tacke, Raphael Mohr

**Affiliations:** 1 Department of Hepatology and Gastroenterology, Charité Universitätsmedizin Berlin, Campus Virchow Klinikum (CVK) and Campus Charité Mitte (CCM), Augustenburger Platz 1, 13353 Berlin, Germany; joeri.lambrecht@charite.de (J.L.); fabian.jost-brinkmann@charite.de (F.J.-B.); muenevver.demir@charite.de (M.D.); raphael.mohr@charite.de (R.M.); 2 Department of Medicine, Ruhr University Bochum, University Hospital Knappschaftskrankenhaus Bochum, In der Schornau 23-25, 44892 Bochum, Germany; Mustafa.Oezcueruemez@kk-bochum.de (M.P.-Ö.); jan.best@med.ovgu.de (J.B.); 3 Clinic for Gastroenterology, Hepatology and Infectious Diseases, University Hospital Düsseldorf, Medical Faculty of Heinrich Heine University Düsseldorf, Moorenstraße 5, 40225 Düsseldorf, Germany; christoph.roderburg@med.uni-duesseldorf.de

**Keywords:** liquid biopsy, GALAD, PDGFRβ, liver cancer, biomarker, cirrhosis

## Abstract

(1) Background: Surveillance of at-risk patients for hepatocellular carcinoma (HCC) is highly necessary, as curative treatment options are only feasible in early disease stages. However, to date, screening of patients with liver cirrhosis for HCC mostly relies on suboptimal ultrasound-mediated evaluation and α-fetoprotein (AFP) measurement. Therefore, we sought to develop a novel and blood-based scoring tool for the identification of early-stage HCC. (2) Methods: Serum samples from 267 patients with liver cirrhosis, including 122 patients with HCC and 145 without, were collected. Expression levels of soluble platelet-derived growth factor receptor beta (sPDGFRβ) and routine clinical parameters were evaluated, and then utilized in logistic regression analysis. (3) Results: We developed a novel serological scoring tool, the APAC score, consisting of the parameters age, sPDGFRβ, AFP, and creatinine, which identified patients with HCC in a cirrhotic population with an AUC of 0.9503, which was significantly better than the GALAD score (AUC: 0.9000, *p* = 0.0031). Moreover, the diagnostic accuracy of the APAC score was independent of disease etiology, including alcohol (AUC: 0.9317), viral infection (AUC: 0.9561), and NAFLD (AUC: 0.9545). For the detection of patients with (very) early (BCLC 0/A) HCC stage or within Milan criteria, the APAC score achieved an AUC of 0.9317 (sensitivity: 85.2%, specificity: 89.2%) and 0.9488 (sensitivity: 91.1%, specificity 85.3%), respectively. (4) Conclusions: The APAC score is a novel and highly accurate serological tool for the identification of HCC, especially for early stages. It is superior to the currently proposed blood-based algorithms, and has the potential to improve surveillance of the at-risk population.

## 1. Introduction

Over the past decades, the incidence of hepatocellular carcinoma (HCC) has been steadily increasing, and only most recently a slight decrease has been observed [1]. HCC is the fifth most common cancer worldwide [2], accounting for one of the leading causes of cancer-related deaths, and therefore, it presents a major global health issue.

The vast majority of HCC develops as a late complication of ongoing liver inflammation and cirrhotic transformation, e.g., due to viral hepatitis, alcohol-related liver damage, and non-alcoholic fatty liver disease (NAFLD) [3]. The tumor stage and degree of liver injury mutually determine the prognosis of patients with HCC, which often remains poor. Patients diagnosed with early-stage HCC, i.e., stage 0 or A by Barcelona Clinic of Liver Cancer (BCLC) staging, may undergo tumor resection or may be considered for liver transplantation in case of limited tumor burden [4]. A 5-year survival rate of 70–75% can be expected from this group. However, many patients are diagnosed with advanced stages of HCC [5]. These patients are left to palliative treatments only and life expectancy is reduced to approximately one year [6,7]. Therefore, surveillance of the at-risk populations is crucial, allowing early diagnosis and improvement of prognosis.

American (AASLD) [8] and European (EASL) [9] guidelines recommend the periodic use of ultrasound scanning (USS), with or without α-fetoprotein (AFP) evaluation, for HCC surveillance. However, suboptimal performance of USS has been reported, with its sensitivity being compromised by the extent of liver cirrhosis, high body mass index (BMI), etiology of liver disease, expertise of the operator and quality of the equipment [10,11]. Moreover, its sensitivity and specificity for early-stage HCC was found to be rather low [12]. In order to overcome these USS-associated limitations, novel serological HCC scoring tools have been proposed, of which the GALAD sore [12], based on gender, age, AFP-L3, AFP, and des-gamma carboxyprothrombin (DCP), was found to have the highest diagnostic potential, as evaluated in multiple international, multicenter, case-control studies [13,14,15,16]. However, as the components of the GALAD score are associated with late-stage HCC characteristics, including the association of DCP levels with portal vein invasion [17] and AFP-L3 levels with tumor metastasis and poor differentiation [18], its value for surveillance of early-stage HCC remains uncertain. The need for a non-invasive tool, suitable for HCC surveillance, regardless of the stage of HCC, thus remains.

The dimeric platelet-derived growth factor receptor beta (PDGFRβ), the cellular receptor for PDGF-BB/AB, is strongly up-regulated during hepatic stellate cell activation upon liver injury [19,20]. Moreover, its expression is strongly increased in the tissue of HCC as compared to the peritumoral tissue [21], and potentially reflects the presence of cancer-associated fibroblasts (CAFs) [22]. PDGFRβ is a key player for the angiogenic and proliferative abilities of HCC and is therapeutically targeted by the multi-tyrosine kinase inhibitors sorafenib and lenvatinib [23]. While circulating soluble PDGFRβ has been recently used for the diagnosis of liver fibrosis independent of disease etiology [19], its diagnostic performance in HCC remains unknown. In this study, using a multi-etiological, multi-stage real-world cohort of patients with liver cirrhosis, we aimed to investigate the diagnostic value of soluble PDGFRβ for HCC, and to compare, develop, and validate novel serological diagnostic HCC scoring-tools.

## 2. Material and Methods

### 2.1. Study Population

This exploratory observational cohort study was performed to evaluate a potential role of soluble PDGFRβ as a diagnostic biomarker for HCC in patients with liver cirrhosis. In total, 267 patients with liver cirrhosis, including 122 patients with HCC, were recruited at the Department of Hepatology and Gastroenterology at Charité Universitätsmedizin Berlin during their regular visits. All etiologies of liver cirrhosis and all BCLC stages of HCC were accepted. Inclusion criteria included: (i) The presence of liver cirrhosis, as determined according to clinical, serological, and radiological findings [24]. (ii) Verified presence or absence of HCC, assessed by computed tomography (CT) and/or magnetic resonance imaging (MRI) or based on histological validation. In patients with presence of liver cirrhosis, non-invasive diagnosis of HCC is standard, when dynamic imaging shows typical diagnostic patterns as the combination of hypervascularity in late arterial phase and washout on portal venous and/or delayed phases. (iii) Availability of complete clinical information. Blood samples for HCC patients were collected at tumor diagnosis and prior to any tumor specific therapy. Samples were centrifuged for 10 min at 2000× *g*, and serum samples were then stored at −80 °C until use. This study was approved by the ethics committee of Charité Berlin, Germany (EA2/091/19) and conducted in accordance with the ethical standards laid down in the Declaration of Helsinki. Written informed consent was obtained from every patient.

### 2.2. Measurement of Serological Markers

Circulating levels of AFP, AFP-L3, and DCP were measured using the μTAS Wako i30 fully automated immunoanalyzer (FUJIFILM Wako Chemicals Europe GmbH, Neuss, Germany). Assay sensitivities were 0.1 ng/mL for DCP and 0.3 ng/mL for AFP. The percentage of AFP-L3 was determined in samples where both subfractions (AFP-L1 and AFP-L3) were >0.3 ng/mL. Soluble PDGFRβ was measured by a commercially available enzyme-linked immunosorbent assay (ELISA) (ThermoFisher Scientific, Waltham, MA, USA), according to manufacturer’s instructions. Serum samples were diluted 1/10 with diluent provided by the manufacturer. Absorbance values were obtained with a SpectraMax i3x microplate reader (Molecular Devices, San Jose, CA, USA).

Diagnostic scoring tools were calculated using the following formulae [25]:

ALBI = (log_10_ bilirubin [µmol/L] × 0.66) + (albumin [g/L] × − 0.085)

Fib-4 = age × AST [IU/L]/(platelet count [10^9^/L] × (ALT [IU/L]^1/2^)

GALAD = (0.09 × age + 1.67 × gender) + (2.34 × log_10_ (AFP [ng/mL])) + (0.04 × AFP-L3 [%]) + (1.33 × log_10_ (DCP [ng/mL])) − 10.08

Gender is set as 1 for female and 0 for male [12].

### 2.3. Statistical Analysis

Data were analyzed using GraphPad Prism 6 (GraphPad, Palo Alto, CA, USA) and MedCalc version 18 (MedCalc Software, Ostend, Belgium) statistical software. Quantitative variables are expressed as median (IQR). Statistical analyses were performed using the Mann–Whitney test and Kruskal–Wallis test with Dunn’s post hoc test, as appropriate. Statistical differences between categorical values were determined using the Chi-square test. The diagnostic performance of the depicted biomarkers or diagnostic scores was determined using receiver operating characteristics (ROC) curves, and the area under the curve (AUC) was calculated. AUC values were compared according to DeLong et al. [26]. The optimal cut-off values, and related sensitivity and specificity, were computed based on the highest Youden’s index [27]. Correlation studies were executed using Spearman’s correlation test. The sufficiency of the sample size was confirmed by MedCalc version 18, using in-house preliminary results, a type I error rate (α) of 5%, and a power (1-β) of 80%.

## 3. Results

### 3.1. Patient Characteristics

This study enrolled 267 patients with liver cirrhosis, including 122 patients with hepatocellular carcinoma (HCC). Clinical and demographic characteristics of the study participants are shown in Table 1. While the HCC participants were, with a median age of 66 years, significantly older than the control population (54 years, *p* < 0.0001), the gender distribution was found to be consistent between both groups (77.9% male HCC subjects vs. 68% male controls). The three major etiologies of liver disease were equally represented in both patient populations (alcohol: 33.6% HCC vs. 35.2% control, viral: 31.1% HCC vs. 20% control, NAFLD: 21.4% HCC vs. 23.4% control). Control subjects suffered from more advanced cirrhosis (*p* < 0.0001), as shown by higher Child-Pugh classification. As expected, the clinical cirrhosis and HCC scoring tools MELD, ALBI, and GALAD significantly distinguished both patient populations.

### 3.2. Performance of sPDGFRβ Levels for HCC Detection

**Figure 1:**
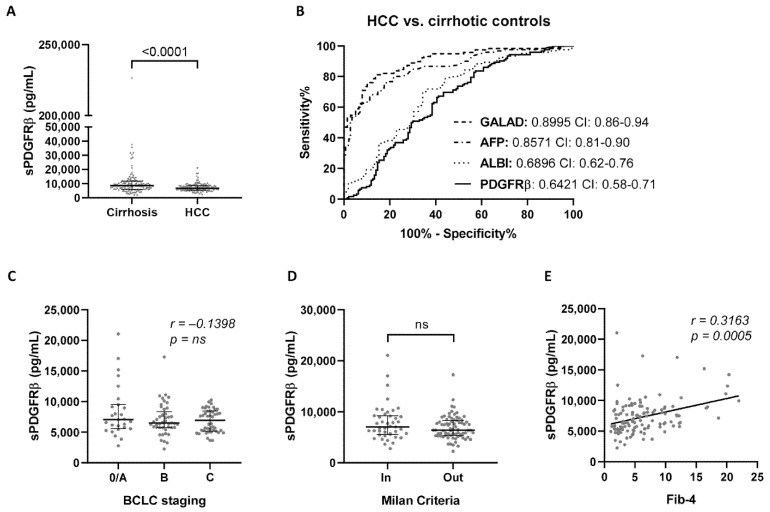
Expression levels of soluble PDGFRβ (sPDGFRβ) in the circulation of cirrhotic patients with (*n* = 122) or without (*n* = 145) HCC. (**A**) Down-regulated expression of circulating sPDGFRβ levels in patients with HCC, as compared to cirrhotic controls. (**B**) Receiver operating characteristic curves for HCC diagnosis, comparing the diagnostic performance of sPDGFRβ to clinical markers. The area under the ROC curve (AUC) values and their confidence interval (CI) are given. No correlation is found between circulating sPDGFRβ levels and the stage of HCC, as measured through (**C**) BCLC staging or (**D**) Milan criteria. (**E**) As expected, in those individuals with HCC, a significant correlation between sPDGFRβ and the stage of fibrosis, as measured via Fib-4, is observed.

Significantly (*p* < 0.0001) lower sPDGFRβ levels are present in the circulation of HCC patients (median (IQR): 6767 (5446–823) pg/mL) compared to cirrhosis control subjects (median (IQR): 8562 (6011–11,724) pg/mL) (Figure 1A); however, the diagnostic accuracy (AUC: 0.6421) was lower than the clinically used HCC scoring algorithms (Figure 1B, Supplementary Table S1). Serum sPDGFRβ levels were not associated with the stage of HCC as determined via BCLC staging (Figure 1C), nor with tumor burden within the Milan criteria (Figure 1D). As expected, a significant correlation (Spearman’s correlation coefficient 0.3163, *p* = 0.0005) between the extent of fibrosis, as determined by Fib-4, and circulating sPDGFRβ levels was observed in HCC subjects (Figure 1E).

### 3.3. The APAC Score as a Superior Diagnostic HCC Test

Based on the difference in sPDGFRβ levels between control and HCC patients, we hypothesized that this marker might be suitable to improve blood-based composite diagnostic tools for HCC. Of the total patient cohort, referred to as the training cohort, 70% was used in the logistic regression analysis to identify HCC-linked parameters, and to combine them into a novel scoring algorithm. Age, sPDGFRβ, AFP, and Creatinine, were identified as the main HCC determinants, and were combined into the APAC score, weighted by their regression coefficients:

APAC score = (Age [years] × 0.20480) − (log_10_(sPDGFRβ [pg/mL]) × 1.98684) + (log_10_(AFP [ng/mL]) × 2.45657) − (Creatinine [mg/dL] × 2.46891) − 4.36493

**Figure 2:**
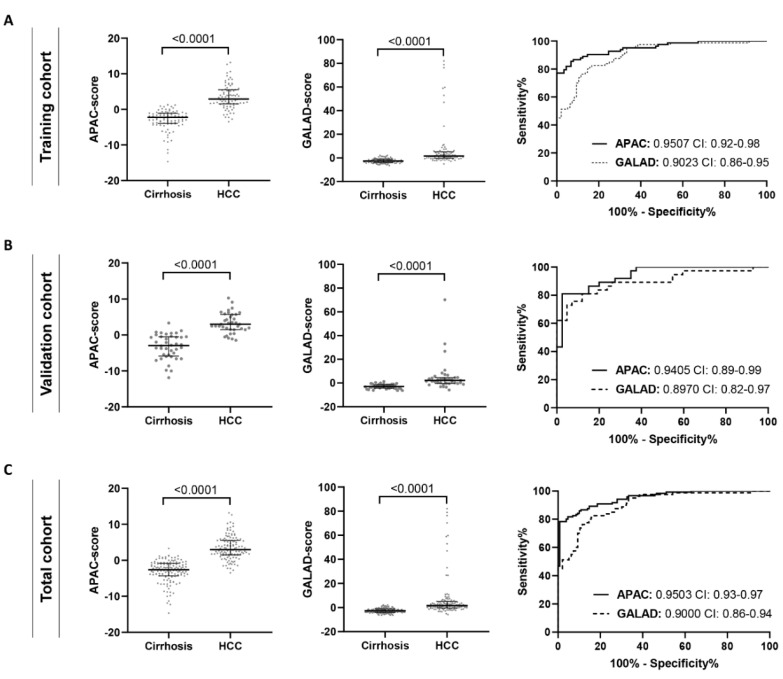
Comparison of the performance of the APAC and GALAD score for the diagnosis of HCC in the (**A**) training cohort, (**B**) validation cohort, and (**C**) total cohort. The area under the ROC curve (AUC) values and their confidence interval (CI) are given.

**Figure 3:**
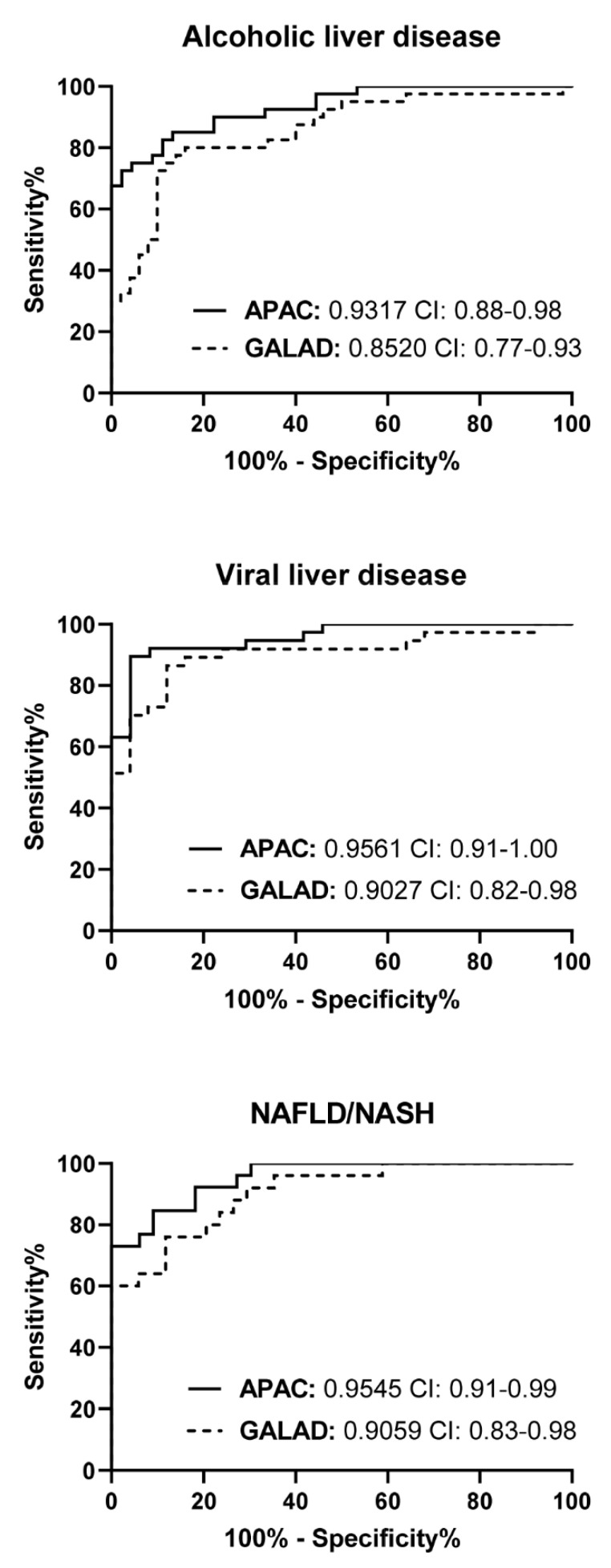
Etiology independence of the APAC score for diagnosis of HCC. Receiver operating characteristic curves identified constant, and superior, diagnostic accuracy of the APAC score for the diagnosis of HCC in each etiology-specific patient sub cohort. The area under the ROC curve (AUC) values and their confidence interval (CI) are given.

The APAC score showed a diagnostic performance (AUC: 0.9507), which was significantly higher than the use of its individual parameters (AUC, sPDGFRβ: 0.6214, AFP: 0.8478, Creatinine: 0.6401) and the GALAD score (AUC: 0.9023) (Figure 2A, Table 2). Diagnostic superiority of the APAC score over the GALAD score was validated in the remaining 30% of the cohort (validation cohort) (AUC, APAC: 0.9405, GALAD: 0.8970) (Figure 2B, Table 2). In the total cohort, using a cut-off value of -0.63, the GALAD score achieved 81.2% sensitivity and 85.5% specificity, the APAC score with cut-off value 0.7969 achieved 81.7% sensitivity and 95.4% specificity (Figure 2C, Table 2). The diagnostic performance of the APAC score is not solely based on the extent of cirrhosis, because its diagnostic value remained superior as compared to the GALAD score, in HCC patients and control subjects in an early Child-Pugh stage (Child-Pugh A) (Supplementary Figure S1). Dividing the total patient population into etiology-specific subgroups demonstrated that the APAC score performs superior for the detection of HCC over individual parameters (Supplementary Table S2, Supplementary Figure S2) and the GALAD score, independent of the underlying etiology of cirrhosis (AUC, alcohol: APAC 0.9317 vs. GALAD 0.8520 (*p* = 0.0499), viral: APAC 0.9561 vs. GALAD 0.9027 (*p* = 0.0683), NAFLD: APAC 0.9545 vs. GALAD 0.9095 (*p* = 0.1531)) (Figure 3, Supplementary Table S2).

### 3.4. The Diagnostic Performance of the APAC Score Is Independent of the Stage of HCC

**Figure 4:**
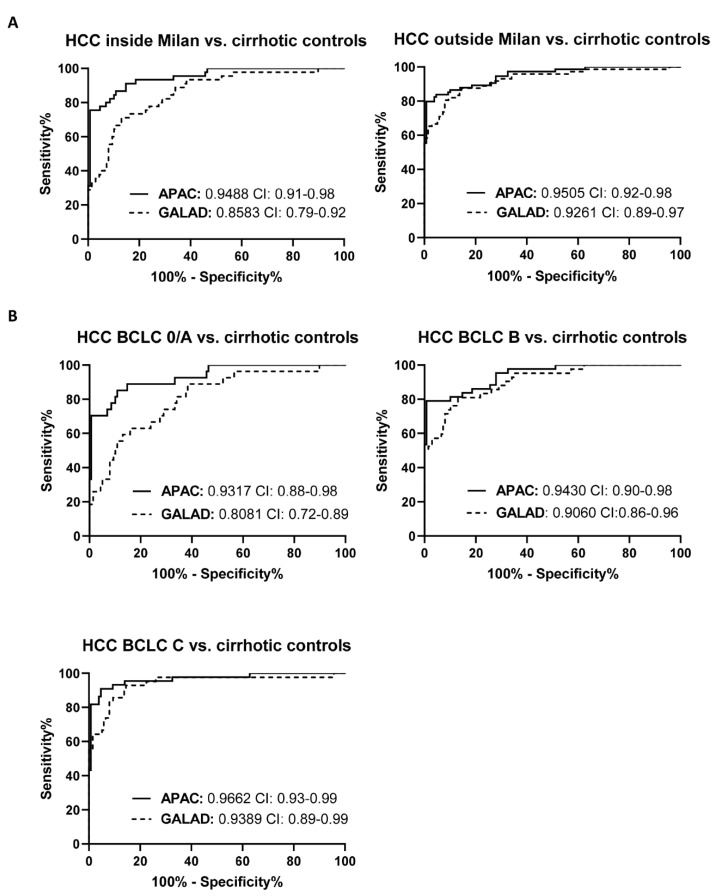
The diagnostic performance of the APAC score is independent of the stage of HCC. (**A**) Construction of the receiver operating characteristics curves identified no difference in diagnostic accuracy of the APAC score for patients inside or outside Milan criteria. (**B**) Moreover, staging of the HCC patients using the BCLC-scoring system identified high and superior diagnostic accuracy of the APAC score for each stage of HCC. The area under the ROC curve (AUC) values and their confidence interval (CI) are given.

Detection of early stages of HCC during surveillance in at-risk patients is of utmost clinical relevance, but remains an unsolved challenge in clinical practice [28]. HCC subjects were divided based on the Milan criteria, which is used to assess eligibility for liver transplantation when suffering from HCC [29]. Significantly higher (*p* = 0.0006) diagnostic performance was observed for the APAC score (AUC: 0.9488, sensitivity: 91.1%, specificity: 85.3%) to identify HCC patients within Milan criteria, as compared to the GALAD score (AUC: 0.8583, sensitivity: 71.1%, specificity: 87.0%) (Figure 4A, Supplementary Table S3). Furthermore, the APAC score was found superior for the identification of HCC patients with early stages (BCLC 0/A), as compared to the GALAD score, with AUCs of, respectively, 0.9317 and 0.8081, *p* = 0.0006 (Figure 4B, Supplementary Table S3). While a significant lower APAC score was observed in patients eligible for liver transplantation compared to those outside Milan criteria, no correlation was found between the APAC score and the BCLC-stage (Supplementary Figure S3). Surprisingly, although both the APAC and GALAD score have AFP as a constitute, the APAC score had higher diagnostic value for the identification of HCC-patients with AFP values lower than 10 or 20 ng/mL (AUC of 0.8780 and 0.8960, respectively), as compared to the GALAD score (AUC of 0.7819 (*p* = 0.0146) and 0.8054 (*p* = 0.0045), respectively) (Supplementary Figure S4, Supplementary Table S4).

## 4. Discussion

In this study, we developed and validated a novel blood-based diagnostic score for HCC, the APAC score, consisting of the demographic/laboratory parameters age, sPDGFRβ, AFP, and creatinine. The APAC score achieved greater diagnostic performance for HCC, and associated higher sensitivity and specificity, as compared to currently proposed scoring tools, including the AFP and the GALAD score. Moreover, compared to these latter tests, the APAC score obtained greater diagnostic value for the identification of patients with (very) early (BCLC 0/A) stage HCC, HCC patients with low AFP values, and those within Milan criteria, therefore suggesting its potential superior performance for surveillance of high-risk individuals. Finally, the APAC score had consistently high and superior diagnostic performance independent of disease etiology, severity of HCC, and extent of cirrhosis.

Due to the fact that most patients are diagnosed with intermediate or advanced stages of HCC, mortality rates remain persistently high. Only in early tumor stages, treatment options with curative intention are applicable. Close surveillance of the at-risk population should be performed, allowing an early identification of HCC development. The reality is, however, that most HCC cases are still diagnosed at advanced stages [28]. Several novel non-invasive diagnostic tests have been developed, of which multiple studies propose the GALAD score, which includes AFP, AFP-L3, DCP, gender, and age, as the scoring tool with the highest possible sensitivity and specificity. Indeed, the high diagnostic performance of the GALAD score has been validated in multiple studies, including large patient cohorts, with various etiologies, stage of HCC, and ethnicities [13,14,15]. One study even identified its diagnostic superiority for HCC detection in a multi-etiological cohort as compared to ultrasound-based diagnosis [13]. The results obtained in our multi-etiological HCC cohort further validate such high diagnostic performance of the GALAD score, obtaining an AUC of 0.8995, with a sensitivity and specificity of, respectively, 81.2% and 85.5%, cut-off = −0.63, which outperformed the MELD score (AUC: 0.7956), ALBI score (AUC: 0.6896), and AFP levels (AUC: 0.8571). However, the diagnostic performance of the GALAD score in our cohort seemed to be lower when compared to previously published data, in which ranging AUC-values are obtained with a maximum AUC of 0.976 [30]. As shown in our analysis, the diagnostic performance of the GALAD score is strongly hampered in early stages (BCLC 0/A) of HCC (AUC: 0.8081), in patients within Milan criteria (AUC: 0.8583), in patients with low (<10 or 20 ng/mL) AFP levels (AUC: 0.7819 and 0.8054, respectively), and in subjects suffering from alcoholic liver disease (AUC: 0.8520). Dependent on the predominant presence of included subjects with these aforementioned characteristics, the diagnostic accuracy of the GALAD score may strongly fluctuate. A more robust scoring system may, therefore, provide better clinical use.

As the extent of cirrhosis has been acknowledged as a significant risk factor for HCC development [31], the use of a biomarker able to represent the extent of fibrosis/cirrhosis may significantly improve the diagnostic value of any future HCC-scoring tool. PDGFRβ has not only been identified as a marker of hepatic stellate cell activation and associated liver fibrosis [19], but has also been found to be elevated in HCC tissue [21], therefore suggesting its ability to mark both cirrhosis and HCC. Interestingly, although the elevated tissue-expression of PDGFRβ upon fibrosis/cirrhosis and HCC, its expression was found to be down-regulated in the circulation of HCC patients, as compared to cirrhotic controls. Nevertheless, logistic regression analysis identified sPDGFRβ as crucial factor for HCC identification, obtaining the highest diagnostic value when combined with age, creatinine, and AFP levels, into the APAC score, able to outperform the GALAD score in any of the mentioned conditions, with particularly better diagnostic performance in patients with early-stage HCC (BCLC 0/A). However, in-depth characterization of circulating sPDGFRβ is necessary to obtain information regarding its stability, mechanisms of release, and clearance, so a fluent integration in routine clinical analysis can be ensured. Since sPDGFRβ most likely adds information on the hepatic stroma to existing tools of HCC diagnosis, it would be interesting to evaluate sPDGFRβ or the APAC score as a predictor and/or indicator of treatment responses in HCC, due to its potential to reflect aspects of the tumor microenvironment.

One additional advantage of the APAC score, besides its superior diagnostic accuracy, concerns its relatively easy and accessible use. Indeed, while creatinine and AFP levels are part of the routine clinical panels, sPDGFRβ can be easily detected and quantified using (commercial) antibody-based systems. This is in strong contrast to the GALAD score, which requires specialized and extremely sensitive (especially for AFP-L3 detection) equipment for analysis, which, therefore, hampers its availability and cost-effectiveness. As both the APAC and GALAD scores are based on the quantification of AFP, it should be noted that fluctuating AFP levels due to flares of viral infection or exacerbation of the underlying liver disease [9] may potentially influence their outcome. However, our results show that the diagnostic value of the APAC score remained high in those HCC patients with low AFP (<10 or 20 ng/mL) values, therefore suggesting sufficient dominant influence by the other factors in such patients with low AFP expression.

Despite the significant clinical value of the reported findings, our study has some limitations. First, most patients included in this study were Caucasian, with only a minor population having Middle Eastern origin. As a significant difference has been observed in incidence of HCC across different ethnicities [32], the evaluation of the APAC score in other ethnicities (e.g., Asian and African) requires further attention. Second, although HCC subjects with stage BCLC 0/A are the targeted screening population, our patient cohort only included a relatively low number (22.1%) of such (very) early-stage HCC individuals. Third, the included patient population solely consisted of patients with cirrhosis, so that an evaluation in other “at-risk” populations (e.g., NAFLD with bridging fibrosis, hepatitis B with a PAGE-B score > 10) is warranted. However, it should be noted that the distribution of our cohort largely reflects real clinical circumstances, as the incidence of HCC is significantly higher in individuals with cirrhosis, compared to those without [31], and are, thus, the target audience of preference for HCC surveillance. Last, validation of the APAC score in an independent, large, and prospectively collected patient cohort is highly desired, as this would allow the validation and/or re-definition of the optimal APAC cut-off value.

In conclusion, we developed a novel and objective diagnostic blood-based tool for HCC in patients with liver cirrhosis, the APAC score, which relies on the expression levels of three circulating markers, combined with age. Not only did the APAC score significantly outperform the GALAD score for the detection of HCC, it was also found to be independent of the tumor burden of HCC, etiology, and stage of cirrhosis. Further validation of its diagnostic character and the evaluation of its prognostic performance should be performed in future international multicenter prospective studies.

## Figures and Tables

**Table 1:** Characteristics of the study participants.

	Cirrhosis (*n* = 145)	HCC (*n* = 122)	*p*-Value
Age, median (IQR), years	54 (47 to 60)	66 (60 to 72)	<0.0001
Gender, *n* (%)			0.07979
Female	46 (31.7%)	27 (22.1%)	
Male	99 (68.3%)	95 (77.9%)	
Etiology, *n* (%)			0.1917
Alcohol	51 (35.2%)	41 (33.6%)	
HBV	11 (7.6%)	11 (9.0%)	
HCV	18 (12.4%)	27 (22.1%)	
NAFLD	34 (23.4%)	26 (21.4%)	
Other	31 (21.4%)	17 (13.9%)	
Child-Pugh class, *n* (%)			<0.0001
A	57 (39.3%)	99 (81.1%)	
B	58 (40.0%)	22 (18.0%)	
C	10 (6.9%)	0	
Laboratory results, median (IQR)			
AST, IU/L	57.0 (18.0 to 85.0)	63.0 (25.0 to 95.5)	0.1132
ALT, IU/L	35.0 (25.0 to 63.5)	42.0 (29.5 to 73.0)	0.0551
ALP, IU/L	135.0 (99.0 to 183.0)	135.0 (98.0 to 200.5)	0.6679
GGT, IU/L	94.0 (50.75 to 205.5)	170.0 (87.00 to 278.5)	0.0001
Total bilirubin, mg/dL	2.0 (1.3 to 4.2)	1.1 (1.3 to 4.2)	<0.0001
Albumin, g/L	33.0 (29.0 to 39.0)	37.1 (33.0 to 40.8)	0.0003
Thrombocytes, ×10^3^/mm^3^	99.0 (70.0 to 149.0)	125.0 (83.0 to 211.5)	0.0059
Creatinine, mg/dL	0.92 (0.76 to 1.21)	0.82 (0.70 to 0.98)	0.0005
INR	1.40 (1.24 to 1.63)	1.18 (1.07 to 1.29)	<0.0001
CRP, mg/L	1.28 (0.40 to 3.14)	2.35 (0.62 to 14.9)	0.0011
AFP, ng/mL	3.5 (2.1 to 6.0)	24.0 (7.1 to 260.7)	<0.0001
AFP-L3, *%*	0.10 (0.10 to 8.10)	14.70 (5.94 to 36.55)	<0.0001
DCP, ng/mL	0.69 (0.27 to 3.89)	6.0 (1.42 to 57.46)	<0.0001
Scoring parameters, median (IQR)			
Fib-4	5.63 (2.95 to 8.59)	5.27 (3.02 to 9.09)	0.9377
MELD	14.10 (11.09 to 19.28)	9.41 (7.75 to 11.58)	<0.0001
ALBI	−1.72 (−2.42 to −1.22)	−2.35 (−2.73 to −1.85)	<0.0001
GALAD	−2.79 (−4.03 to −1.230)	1.56 (−0.21 to 4.99)	<0.0001
Tumor size, *n* (%)			
≤2 cm	N.A.	14 (11.5%)	N.A.
>2 to ≤3 cm	N.A.	19 (15.6%)	N.A.
>3 to ≤5 cm	N.A.	31 (25.4%)	N.A.
>5 cm	N.A.	42 (34.4%)	N.A.
Tumor number, *n* (%)			
1	N.A.	45 (36.9%)	N.A.
2	N.A.	18 (14.8%)	N.A.
≥3	N.A.	56 (45.9%)	N.A.
BCLC stage, *n* (%)			
Very early (0)	N.A.	7 (5.7%)	N.A.
Early (A)	N.A.	20 (16.4%)	N.A.
Intermediate (B)	N.A.	44 (36.1%)	N.A.
Advanced (C)	N.A.	44 (36.1%)	N.A.
Milan Criteria, *n* (%)			
Inside	N.A.	75 (61.5%)	N.A.
Outside	N.A.	43 (35.2%)	N.A.

ALBI, albumin-bilirubin score; AFP, α-fetoprotein; ALT, alanine aminotransferase; ALP, alkaline phosphatase; AST, aspartate aminotransferase; BCLC, Barcelona clinic liver cancer; CRP, C-reactive protein; DCP, des-gamma carboxyprothrombin; Fib-4, fibrosis-4 score; GALAD, gender, age, AFP-L3, and DCP score; GGT, gamma-glutamyl transferase; HBV, hepatitis B virus; HCV, hepatitis C virus; HCC, hepatocellular carcinoma; INR, international normalized ratio; NA, not applicable; NAFLD, non-alcoholic fatty liver disease; ns, non-significant; MELD, model of end stage liver disease.

**Table 2:** Accuracy of hepatocellular carcinoma diagnosis in the training, validation, and total cohort using the APAC score, in comparison to its constitutes and the GALAD score.

	Cut-Off Value	AUC (95% CI)	*p*-Value AUC (vs. APAC)	Sensitivity, %	Specificity, %	PPV, %	NPV, %
**Training cohort**							
APAC	0.4109	0.9507 (0.9202–0.9813)	-	85.54	93.26	91.43	88.39
sPDGFRβ, pg/mL	7962	0.6214 (0.5414–0.7015)	<0.0001	68.24	54.46	55.76	67.09
Creatinine, mg/dL	1.025	0.6401 (0.5584–0.7218)	<0.0001	83.33	42.22	54.82	75.07
AFP, ng/mL	9.800	0.8478 (0.7920–0.9037)	0.0001	65.06	90.00	84.55	75.38
GALAD	−0.8141	0.9023 (0.8584–0.9463)	0.0252	82.50	83.33	80.63	84.98
**Validation cohort**							
APAC	0.6771	0.9405 (0.8920–0.9891)	-	81.08	92.5	90.09	85.32
sPDGFRβ, pg/mL	10155	0.7039 (0.5892–0.8187)	0.0006	83.78	56.82	62.01	80.64
Creatinine, mg/dL	0.9050	0.5923 (0.4670–0.7177)	<0.0001	64.86	54.76	54.76	64.94
AFP, ng/mL	12.95	0.8826 (0.8032–0.9619)	0.0182	72.97	95.24	92.80	80.73
GALAD	−0.5396	0.8970 (0.8231–0.9710)	0.0137	81.08	88.1	85.15	84.69
**Total cohort**							
APAC	0.7969	0.9503 (0.9258–0.9747)	-	81.67	95.35	93.66	86.08
sPDGFRβ, pg/mL	9278	0.6470 (0.5813–0.7127)	<0.0001	83.61	43.45	55.43	75.91
Creatinine, mg/dL	0.9550	0.6266 (0.5583–0.6948)	<0.0001	72.73	49.24	54.66	68.22
AFP, ng/mL	6.350	0.8571 (0.8113–0.9028)	<0.0001	79.17	78.87	75.92	81.82
GALAD	−0.6373	0.9000 (0.8620–0.9380)	0.0031	81.20	85.51	82.50	84.39

AFP, alpha-fetoprotein; APAC, age, PDGFRβ, AFP, creatinine score; AUC, area under the ROC curve; GALAD, gender, age, AFP-L3, and DCP score; PDGFRβ, platelet derived growth factor receptor beta, PPV, positive predictive value; NPV, negative predictive value.

## Data Availability

The data presented in this study are available on request from the corresponding author.

## Supplementary Materials

The following material is available online at https://www.mdpi.com/article/10.3390/jcm10153392/s1: Figure S1: The diagnostic accuracy of the APAC-score is not influenced by the extent of fibrosis, Figure S2: Etiology-independence of the APAC-score, compared to its constitutes, for diagnosis of HCC, Figure S3: Relationship of the APAC-score with the stage of HCC, as measured through Milan criteria and BCLC-staging, Figure S4: Diagnostic utility of the APAC-score in HCC patients with low levels of AFP, Table S1: accuracy of soluble PDGFRβ-levels for the diagnosis of hepatocellular carcinoma, in comparison to clinical-used serological HCC scoring tools, Table S2: Comparison of the performance of the APAC-score with its constitutes and the GALAD score for diagnosis of HCC in etiology-specific cohorts, Table S3: Comparison of the performance of the APAC-score with the GALAD score for diagnosis of early or late stage HCC, Table S4: Performance of the APAC-score, in comparison to the GALAD-score, for diagnosis of HCC in patients with high or low levels of AFP.

## Abbreviations

AASLD: American association for the study of the liver; AFP, alpha-fetoprotein; ALBI, albumin-bilirubin score; APAC, age, sPDGFRβ, AFP, and creatinine score; AUC, area under the curve; BCLC, Barcelona clinic liver cancer-staging; BMI, body mass index; CAFs, cancer-associated fibroblasts; CI, confidence interval; DCP, des-gamma carboxyprothrombin; EASL, European association for the study of the liver; ELISA, enzyme-linked immunosorbent assay; Fib-4, fibrosis-4 score; GALAD, gender, age, AFP-L3, AFP, and des-gamma carboxyprothrombin score; HCC, hepatocellular carcinoma; IQR, interquartile range; MELD, model of end stage liver disease; NAFLD, non-alcoholic fatty liver disease; PDGF, platelet derived growth factor; ROC, receiver operating characteristics; sPDGFRβ, soluble platelet derived growth factor receptor beta; USS, ultrasound scanning.
