# Supplementary material for: The APAC Score: A Novel and Highly Performant Serological Tool for Early Diagnosis of Hepatocellular Carcinoma in Patients with Liver Cirrhosis
Source: J Clin Med. 2021 Jul 30;10(15):3392. doi: 10.3390/jcm10153392 (PMC8348918; doi:10.3390/jcm10153392)
Supplement: Supplementary file 1 [file jcm-10-03392-s001.zip › jcm-1326131-supplementary.pdf]

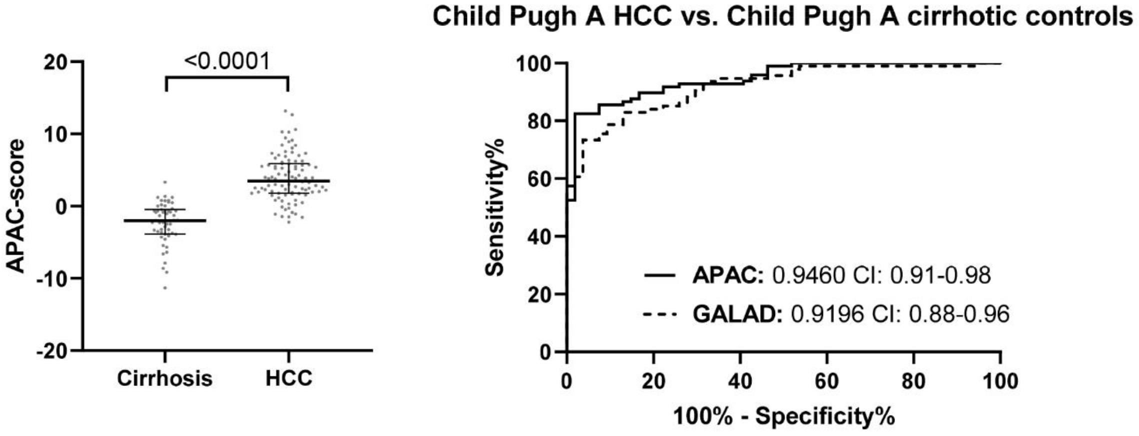

Supplementary Figure S1. The diagnostic accuracy of the APAC-score is not influenced by the extent of cirrhosis. The comparison of APAC-scores between HCC patients and cirrhotic controls, with all individuals categorized into Child-Pugh stage A, identified the high and superior diagnostic accuracy of the APAC-score, as compared to the GALAD-score. The area under the ROC-curve (AUC) values and their confidence interval (CI) are given.

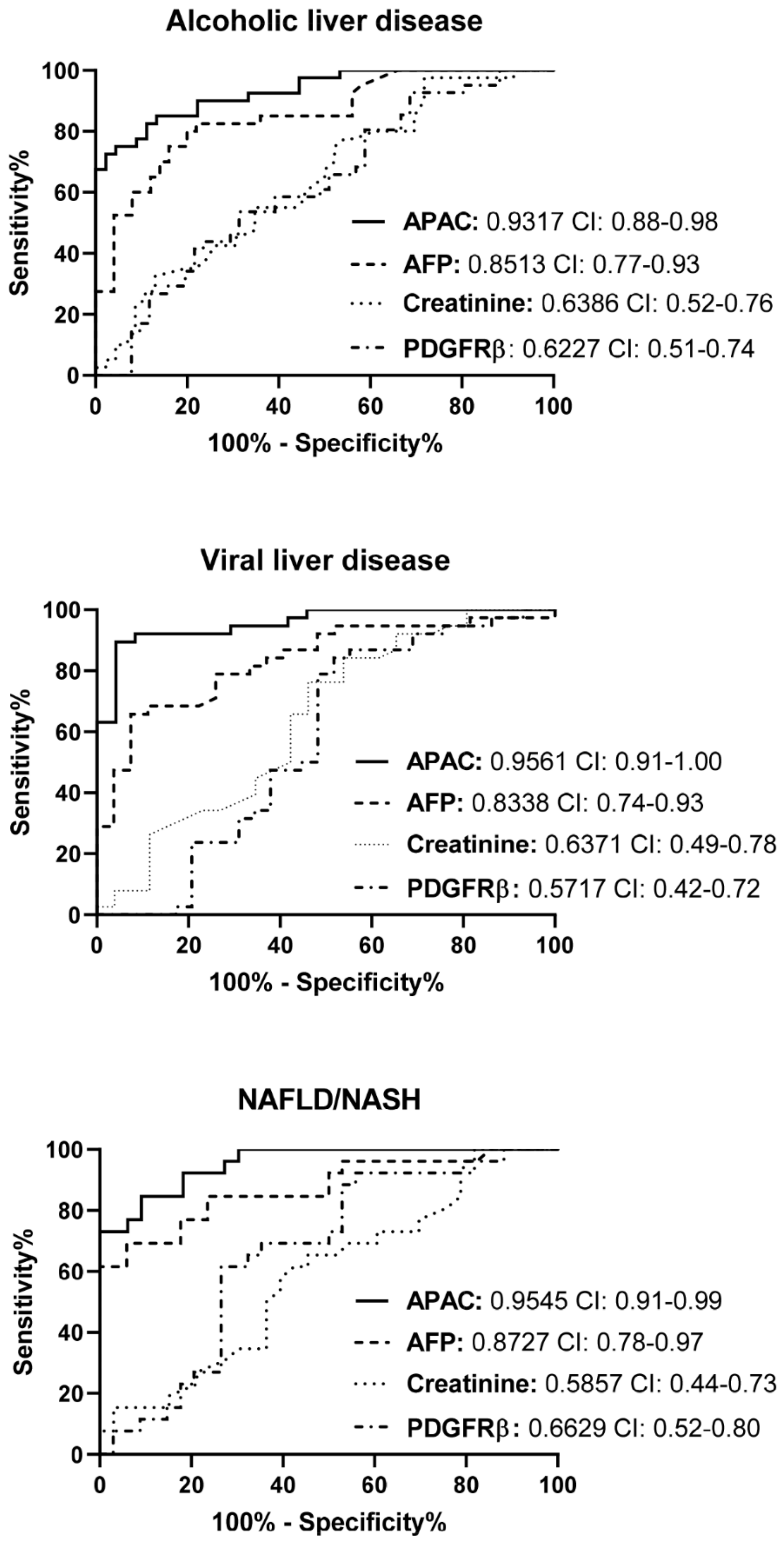

Supplementary Figure S2. Etiology-independence of the APAC-score, compared to it constitutes, for diagnosis of HCC. Receiver operating characteristic curves identified constant, and superior, diagnostic accuracy of the APAC-score for the diagnosis of HCC in each

etiology-specific HCC patient cohort. The area under the ROC-curve (AUC) values and their confidence interval (CI) are given.

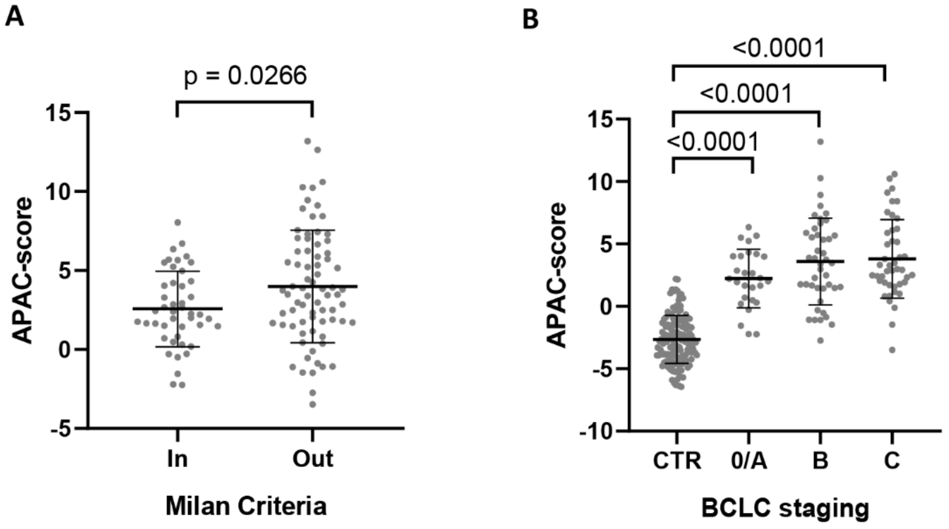

Supplementary Figure S3. Relationship of the APAC-score with the stage of HCC, as measured through (A) Milan criteria and (B) BCLC-staging.

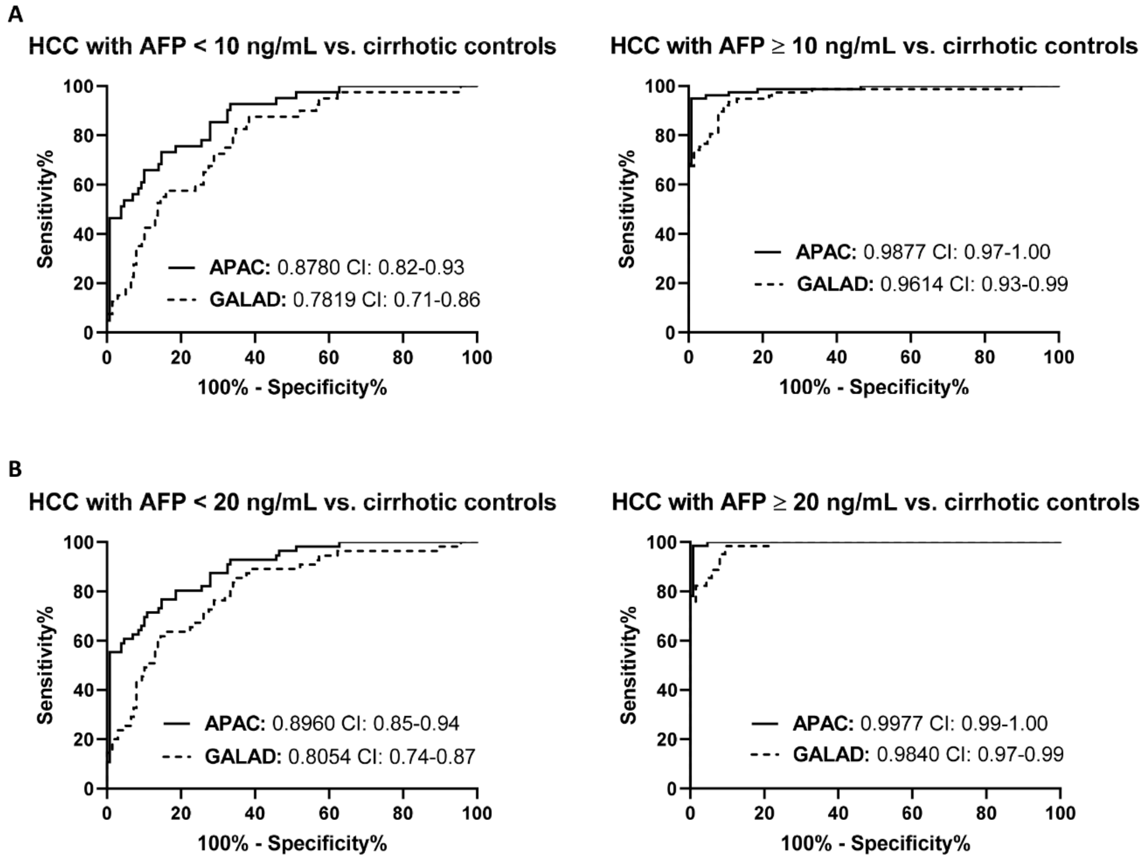

Supplementary Figure S4. Diagnostic utility of the APAC-score in HCC patients with low levels of AFP. HCC patients were divided into those with low and high levels of circulating AFP, based on the cut-offs of (A) 10 ng/mL and (B) 20 ng/mL. Receiver operating characteristic curves identified the APAC-score to be superior for HCC diagnosis, independent of the circulating AFP levels. The area under the ROC-curve (AUC) values and their confidence interval (CI) are given.

Supplementary Table S1. Accuracy of soluble PDGFRβ-levels for the diagnosis of hepatocellular carcinoma, in comparison to clinical-used serological HCC scoring tools.

	**Cut-off value**	**AUC (95% CI)**	**p-value AUC (vs. sPDGFRβ)**	**Sensitivity, %**	**Specificity, %**	**PPV, %**	**NPV, %**
sPDGFRβ, *pg/mL*	8106	0.6421 (0.5758-0.7084)	-	69.67	56.64	57.48	68.94
AFP, *ng/mL*	6.350	0.8571 (0.8113-0.9028)	< 0.0001	79.17	78.17	75.31	81.69
MELD	11.06	0.7956 (0.7390-0.8521)	0.0075	72.73	76.03	71.85	76.82
ALBI	-1.999	0.6896 (0.6195-0.7598)	ns	70.25	65.71	63.28	72.42
GALAD	-0.6373	0.8995 (0.5612-0.9379)	< 0.0001	81.20	85.51	82.50	84.39

AFP, alpha-fetoprotein; ALBI, albumin-bilirubin score; APAC, age, PDGFRβ, AFP, creatinine-score; AUC, area under the ROC curve; GALAD, gender, age, AFP-L3, and DCP-score; MELD, model for end-stage liver disease; NPV, negative predictive value; PDGFRβ, platelet derived growth factor receptor beta, PPV, positive predictive value;.

Supplementary Table S2. Comparison of the performance of the APAC-score with its constitutes and the GALAD score for diagnosis of hepatocellular carcinoma in etiology-specific cohorts.

	Cut-off value	AUC (95% CI)	p-value AUC (vs. APAC)	Sensitivity, %	Specificity, %	PPV, %	NPV, %
**Alcoholic liver disease**							
APAC	0.6419	0.9317 (0.8811-0.9822)	-	75.00	95.56	93.14	82.63
sPDGFRβ, *pg/mL*	11228	0.6227 (0.5084-0.7370)	< 0.0001	92.68	31.37	52.05	84.21
Creatinine, *mg/dL*	1.450	0.6386 (0.5222-0.7550)	< 0.0001	97.50	28.26	52.21	52.21
AFP, *ng/mL*	6.050	0.8513 (0.7723-0.9302)	0.0500	82.50	78.00	75.09	75.09
GALAD	-0.4254	0.8520 (0.7704-0.9336)	0.0499	80.00	84.00	80.07	83.94
**Viral liver disease**							
APAC	0.7731	0.9561 (0.9096-1.0000)	-	92.11	91.67	93.54	93.54
sPDGFRβ, *pg/mL*	8907	0.5717 (0.4201-0.7233)	0.0002	84.21	48.28	68.08	70.01
Creatinine, *mg/dL*	0.9150	0.6371 (0.4922-0.7821)	0.0001	84.21	46.15	67.19	69.05
AFP, *ng/mL*	23.45	0.8338 (0.7351-0.9325)	0.0005	65.79	92.59	92.08	67.38
GALAD	-1.018	0.9027 (0.8242-0.9812)	0.0683 (ns)	86.49	88.00	90.42	83.26
**NAFLD**							
APAC	-0.1217	0.9545 (0.9094-0.9997)	-	84.62	90.91	87.68	88.55
sPDGFRβ, *pg/mL*	9731	0.6629 (0.5236-0.8021)	0.0001	92.31	44.12	55.81	88.24
Creatinine, *mg/dL*	1.090	0.5857 (0.4392-0.7321)	< 0.0001	65.38	54.55	52.38	67.33
AFP, *ng/mL*	9.750	0.8727 (0.7788-0.9666)	0.0286	69.23	94.12	90.00	80.00
GALAD	-0.8344	0.9095 (0.8322-0.9796)	0.1531 (ns)	76.00	88.24	83.17	82.78

AFP, alpha-fetoprotein; APAC, age, PDGFRβ, AFP, creatinine-score; AUC, area under the ROC curve; GALAD, gender, age, AFP-L3, and DCP-score; NPV, negative predictive value; ns, non-significant; PDGFRβ, platelet derived growth factor receptor beta; PPV, positive predictive value.

Supplementary Table S3. Comparison of the performance of the APAC-score with the GALAD score for diagnosis of early or late stage hepatocellular carcinoma.

	Cut-off value	AUC (95% CI)	p-value AUC (vs. APAC)	Sensitivity, %	Specificity, %	PPV, %	NPV, %
**Inside Milan criteria**							
APAC	-0.3225	0.9488 (0.9133-0.9844)	-	91.11	85.27	64.71	97.00
GALAD	-0.4405	0.8583 (0.7961-0.9205)	0.0006	71.11	86.96	61.79	91.03
**Outside Milan criteria**							
APAC	0.7969	0.9505 (0.9205-0.9804)	-	83.78	95.35	90.31	91.91
GALAD	0.0972	0.9261 (0.8857-0.9666)	0.1899 (ns)	80.56	92.03	83.94	90.15
**BCLC 0/A**							
APAC	0.1839	0.9317 (0.8787-0.9846)	-	85.19	89.15	59.37	97.00
GALAD	-2.2860	0.8081 (0.7195-0.8967)	0.0006	88.89	61.59	30.10	96.75
**BCLC B**							
APAC	1.3970	0.9430 (0.9047-0.9813)	-	79.07	99.22	96.85	93.98
GALAD	-0.4405	0.9060 (0.8550-0.9570)	0.0754 (ns)	80.95	86.96	65.32	93.77
**BCLC C**							
APAC	0.7969	0.9662 (0.9332-0.9991)	-	90.91	95.35	85.58	97.19
GALAD	-0.6373	0.9389 (0.8900-0.9879)	0.2570 (ns)	92.86	85.51	66.03	97.53

APAC, age, PDGFRβ, AFP, creatinine-score; AUC, area under the ROC curve; BCLC, Barcelona clinic liver cancer-score; GALAD, gender, age, AFP-L3, and DCP-score; ns, non-significant; NPV, negative predictive value; PPV, positive predictive value.

Supplementary Table S4. Performance of the APAC-score, in comparison to the GALAD-score, for diagnosis of hepatocellular carcinoma in patients with high or low levels of AFP.

	AUC (95% CI)	p-value AUC (vs. APAC)
**AFP < 10 ng/mL**		
APAC	0.8780 (0.8212-0.9349)	-
GALAD	0.7819 (0.7052-0.8586)	0.0146
**AFP ≥ 10 ng/mL**		
APAC	0.9877 (0.9741-1.0000)	-
GALAD	0.9614 (0.9336-0.9893)	0.0126
**AFP < 20 ng/mL**		
APAC	0.8960 (0.8487-0.9434)	-
GALAD	0.8054 (0.7375-0.8733)	0.0045
**AFP ≥ 20 ng/mL**		
APAC	0.9977 (0.9939-1.0000)	-
GALAD	0.9840 (0.9716-0.9964)	0.0367

AFP, alpha-fetoprotein; APAC, age, PDGFRβ, AFP, creatinine-score; AUC, area under the ROC curve; GALAD, gender, age, AFP-L3, and DCP-score; ns, non-significant; NPV, negative predictive value; PPV, positive predictive value.
